# Temporal Patterns in the Abundance of a Critically Endangered Marsupial Relates to Disturbance by Roads and Agriculture

**DOI:** 10.1371/journal.pone.0160790

**Published:** 2016-08-08

**Authors:** Georgina J. Yeatman, Adrian F. Wayne, Harriet R. Mills, Jane Prince

**Affiliations:** 1 School of Animal Biology, Faculty of Science, University of Western Australia, Crawley, Western Australia, Australia; 2 Department of Parks and Wildlife, Manjimup, Western Australia, Australia; Australian National University, AUSTRALIA

## Abstract

The aim of this study was to investigate how landscape disturbance associated with roads, agriculture and forestry influenced temporal patterns in woylie (*Bettongia penicillata*) abundance before, during and after periods of rapid population change. Data were collected from an area of approximately 140,000 ha of forest within the Upper Warren region in south-western Australia. Woylie abundance was measured using cage trapping at 22 grid and five transect locations with varying degrees of landscape disturbance between 1994 and 2012. We found evidence that the distribution and abundance of woylies over time appears to be related to the degree of fragmentation by roads and proximity to agriculture. Sites furthest from agriculture supported a greater abundance of woylies and had slower rates of population decline. Sites with fewer roads had a greater abundance of woylies generally and a greater rate of increase in abundance after the implementation of invasive predator control. The results of this study suggest that landscape disturbance is less important at peak population densities, but during times of environmental and population change, sites less dissected by roads and agriculture better support woylie populations. This may be due to the role these factors play in increasing the vulnerability of woylies to introduced predators, population fragmentation, weed species invasion, mortality from road collisions or a reduction in available habitat. Strategies that reduce the impact of disturbance on woylie populations could include the rationalisation of forest tracks and consolidation of contiguous habitat through the acquisition of private property. Reducing the impact of disturbance in the Upper Warren region could improve the resilience of this critically important woylie population during future environmental change.

## Introduction

Landscape disturbance associated with multi-use systems is an important consideration for conservation managers. The disturbance of natural habitats can be in the form of habitat modification, fragmentation or the influx of non-native species [1–3]. This study explored whether landscape attributes indicative of disturbance from agriculture, roads and timber harvesting explained temporal and spatial patterns in woylie (*Bettongia penicillata*) abundance from data collected through long term population monitoring. The woylie has a long history of conservation intervention, has recently suffered a sudden and dramatic decline and investigation into the factors associated with its distribution will directly impact the development of conservation actions used for the recovery of the species [4].

Land cleared for agriculture can inhibit the connectivity of mammal populations [5–7] and can increase the abundance of invasive predator species such as foxes (*Vulpes vulpes*) and cats (*Felis catus*) in adjacent forests [8, 9]. The abundance of foxes and cats (both significant predators of woylies [8, 10]) may also relate to the degree of fragmentation by roads [2, 11–14]. Timber harvesting, which is commonly managed in Australian forest systems [15–17], has been shown to have a variety of impacts on marsupial species [18–20] and some evidence suggests that woylies may be afforded some protection from decline processes in more recently harvested areas [21, 22]. Disturbance from roads, agriculture or timber harvesting may not operate independently, for example, roads are more likely to be associated with agricultural land or forests subject to timber harvesting, than land allocated for the conservation of flora and fauna (e.g. national parks; [23–25]).

This study was undertaken as part of a larger framework designed to diagnose the potential causes of the recent woylie decline. Historically the woylie was distributed across much of central, southern and western Australia but by the 1960s, had been restricted to three isolated areas in the south-west of Western Australia [26, 27]. Subsequent conservation efforts led to the removal of the species from the threatened fauna list in 1996 [28]. Having made a substantial recovery to around 200,000 individuals across more than 20 sites [4, 27], there has been a rapid and substantial decline in the abundance of woylie populations resulting in a net loss of 95% between 2002 and 2010 that has been subsequently sustained [4, 10]. The woylie was consequently relisted as critically endangered on the IUCN Redlist [10]. Research topics to date investigating the causative factors in the decline include the impact of predation by introduced foxes and cats [8], population genetics [29], disease [30–34] and food resources [35]. Characterising the nature of population change over space and time and the variables associated with these changes is a key aspect to diagnosing the decline and recovering the species [4, 10].

We used regional data on woylie abundance collected over two decades to explore the possible relationships between landscape disturbance and woylie abundance. If, for example, the relationship between woylie abundance and disturbance varies before versus after the population decline, this might provide some insight into the likely factors driving the decline. This information will have a direct impact on the development of conservation actions for the woylie as it may assist in identifying potential threats to the persistence of the species and the characterisation of sites that support woylie populations.

## Materials and Methods

### Study area

**Fig 1:**
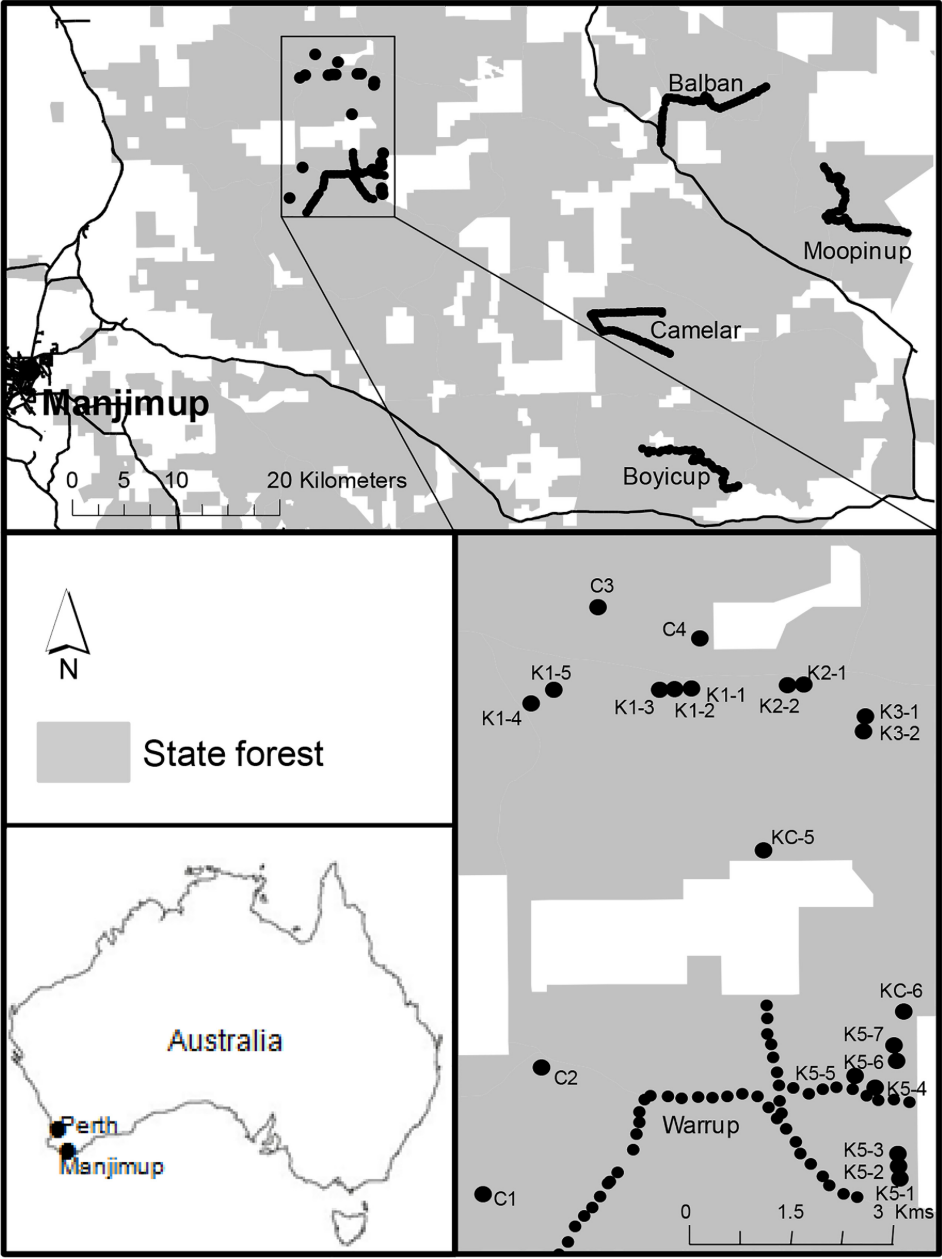
Location of 22 grids and five transects where woylie monitoring was conducted in the Upper Warren, south-western Australia, between 1994 and 2012. Grey shading represents state owned forest managed by the Western Australian Department of Parks and Wildlife. White shading represents privately owned land used for livestock farming and forestry.

The study area in south-western Australia, the Upper Warren, includes approximately 140,000 ha of native forest managed by the Department of Parks and Wildlife (Fig 1). This region is centrally important to the conservation of woylies as it supports one of only two remaining indigenous populations, the extant population is large [4, 28] and it includes two genetically distinct populations that constitute a substantial proportion of the genetic diversity of the species [29]. The study area is bordered by privately owned land predominantly used for livestock farming and forestry. The region has a Mediterranean-type climate with warm dry summers and cool wet winters. The area is characterised by open dry sclerophyll forest and woodlands in which the overstorey is dominated by jarrah (*Eucalyptus marginata*) and marri (*Corymbia calophylla*), and in some places, wandoo (*Eucalyptus wandoo*). The topography is of a gently undulating plateau, with low lateritic ridges and broad valleys [4, 36]. A number of disturbance processes are managed by state authorities within the region including a fuel-reduction burning program, timber harvesting, baiting for the control of introduced red foxes and the control of the plant pathogen *Phytophthora cinnamomi* [4].

### Woylie data collection

Trapping was conducted yearly between 1994 and 2012 as part of the monitoring of medium sized mammalian fauna in the state managed Nature Reserves and National Parks within the Upper Warren. Methodology for trapping woylies was not consistent over the study period as traps were set in either grid or transect formation and the number of traps set each year was variable (S1 Table). At grid sites between 1994 and 2005, traps were set for three consecutive nights during each trapping session. After 2005, this was expanded to four consecutive nights. Traps were set for four consecutive nights during each trapping session at all transect sites across all sampled years. The number of trapping sessions conducted at each site (grid or transect) varied between years. There were between two and six sessions conducted each year at grid sites and one to two sessions per year at transect sites. There were 22 grid sites each consisting of three lines of three traps spaced 80 m apart (total of 9 traps in a 160 m x 160 m square). The five transects consisted of 50 traps spaced 200 m apart (Fig 1). Although there were data available from additional monitoring transects in the Upper Warren [8, 10], only five were selected in order to have continuity of data over years for all transects analysed. Further detail of the methodology of grid and transect monitoring can be found in Wayne *et al*. [8, 10, 22]. All procedures were conducted following guidelines set out by the state government authority at the time but these procedures were modified to improve animal welfare over time. All trapping was conducted with appropriate permits and approved by the appropriate departmental animal ethics committee (Conservation and Land Management, Department of Environment and Conservation or Department of Parks and Wildlife animal ethics committees). The traps used remained consistent over the survey period (hessian-covered wire cages (20 cm x 20 cm x 45 cm), with ‘universal’ bait (peanut butter, rolled oats and sardines). Captured individuals were uniquely marked for identity purposes (Monel 1005–1 ear tags, National Tag and Brand Co., Newport, KY, USA) and standardised data and sample-collection methods were used.

### Landscape disturbance attributes

The landscape attributes selected for analysis were timber harvesting history, road network density and proximity to agriculture. These three variables can be indicative of the degree of disturbance caused by fragmentation and loss of continuity of natural habitat. The number of years since timber harvesting was calculated at each trap point from records kept by the Department of Parks and Wildlife and monitoring of the authorised harvesting activity in the region. Road network density was calculated as the total length of all roads (including sealed and unsealed dual lane public roads and smaller forestry and farm tracks) within a 500 m radius of each trap point. The distance (m) to the nearest agricultural land and the road network density were calculated for each trap point using ArcMap software [37].

### Analyses

We used the percentage of traps that captured a woylie (capture rate) as an index of woylie abundance. Woylie capture rate has been shown to be highly correlated with woylie abundance in the Upper Warren [4, 22]. Woylie abundance and landscape disturbance were averaged across the nine trap points within each grid. Landscape disturbance and woylie abundance were calculated for each trap point along each transect. As part of the exploration of the possible relationships between woylie abundance and landscape disturbance, regression analyses were conducted between each disturbance variable and woylie abundance before, during and after population decline. The categorisation of samples by decline status (before, during or after decline) was estimated for each site independently. Before population decline was classified as data collected during an increasing trend but before peak abundance. During population decline was classified as data collected during a decreasing trend but before lowest abundance levels were reached. After decline was classified as data collected when abundance was no longer declining and had stabilised at relatively low levels.

To understand how woylie abundance related to landscape disturbance over space and time, we categorised sites based on their level of disturbance and then compared the profile of the abundance curves (i.e. abundance trends over time) and absolute values of abundance of each category over time. Grids and transects were analysed separately because of the variation in sampling technique and differences in years of sampling. Sites were classified as high, intermediate and low level disturbance based on road network density, proximity to agriculture and time since timber harvesting. As a result of the available range of variables and in order to try to maintain a similar number of sites in each disturbance level category, the classification of each category varied between grids and transects (Table 1). The classification of disturbance level was made on a scale relative to the available range for grids and transects separately. For example, grid sites in general were much closer to agriculture than transects and so some grid sites that were classified as low disturbance from agriculture would fit into the high disturbance category at transect sites. Harvesting events at some of the grid locations in 1995 and 1996 meant that the classification of some sites changed from ‘Low disturbance’, i.e. long unharvested, to ‘High disturbance’ after being harvested. As a result, the comparison of disturbance from timber harvesting had to be conducted separately in 1994 and 1995. From 1996 to 2009 the classification of grid sites remained consistent and time since timber harvest increased linearly. At transect sites, time since timber harvesting was calculated from 2001 data and increased linearly over time.

**Table 1:** Categories of disturbance at grid and transect locations based on road density, proximity to agriculture and time since timber harvesting.

Disturbance	High disturbance	Intermediate disturbance	Low disturbance
**Road density**			
*Grids*	> 2400 m (6)	1600–2400 m (6)	< 1500 m (6)
*Transects*			
Boyicup, Camelar and Moopinup	> 1800 m (53)	1200–1800 m (50)	< 1200 m (40)
Warrup	> 2500 m (13)	1500–2000 m (10)	< 1500 m (12)
Balban	> 2000 m (17)	1500–2000 m (16)	< 1500 m (16)
**Proximity to agriculture**			
*Grids*	< 300 m (8)	400–810 m (6)	> 900 m (4)
*Transects*			
Boyicup, Camelar and Moopinup	< 1000 m (49)	1000–2000 m (47)	> 2000 m (47)
Warrup	< 1200 m (15)	1200–1800 m (22)	> 1800 m (13)
Balban	< 1000 m (22)	1000–2000 m (18)	> 2000 m (11)
**Time since timber harvesting**			
*Grids*			
1994	-	21–24 yrs (10)	44 yrs (8)
1995	0 yrs (9)	22–25 yrs (6)	45 (3)
1996–2009	0–1 yrs (11)	26 yrs (5)	46 (2)
*Transects*			
Boyicup, Camelar and Moopinup	-	-	-
Warrup	-	22 yrs (16)	42–52 yrs (29)
Balban	-	-	-

Four of the grid locations (C1, K2-2, K3-1 and K3-2) were not used in the analyses as they did not fit a similar abundance pattern over time as the other 18 grids. Boyicup, Camelar and Moopinup transects were analysed as a group and Warrup and Balban transects were analysed separately as they appeared to be experiencing different stages of decline. When comparing disturbance from timber harvesting at grid sites, data from 1994 and 1995 were compared separately to data from 1996–2009. There was no variation in time since timber harvesting for Boyicup, Camelar, Moopinup or Balban and so this attribute was not compared at these sites. Numbers in parentheses indicate the number of sites allocated to that category.

To help visualise the characteristics of sites where woylies were abundant versus sites where woylies were scarce overtime, we conducted a principal coordinate analysis (PCO) of the disturbance variables at each trap location and overlaid vectors indicating woylie abundance. This was conducted on the grid and transects separately in the PERMANOVA+ add-on in the PRIMER-E software package [38], based on Euclidian distances calculated from square-root transformed values.

## Results

A total of 40,664 trap nights between 1994 and 2012 were analysed and recorded 9,530 woylie captures. Correlations between disturbance factors suggested that sites with fewer roads tended to be further from agriculture and have a longer time since timber harvesting. Regression analyses provided no evidence of a relationship between any of the disturbance factors and woylie capture rate before and after population decline, but all three disturbance factors had a significant relationship with woylie capture rate during the population decline (road density P = 0.0006, proximity to agriculture P = 0.002 and time since timber harvesting P = 0.00017). During the decline, sites that caught the most woylies tended to be further from agriculture, have a longer time since timber harvesting and have fewer roads. See S1 Fig and S2 Table for further detail of regression analyses and correlations.

**Fig 2:**
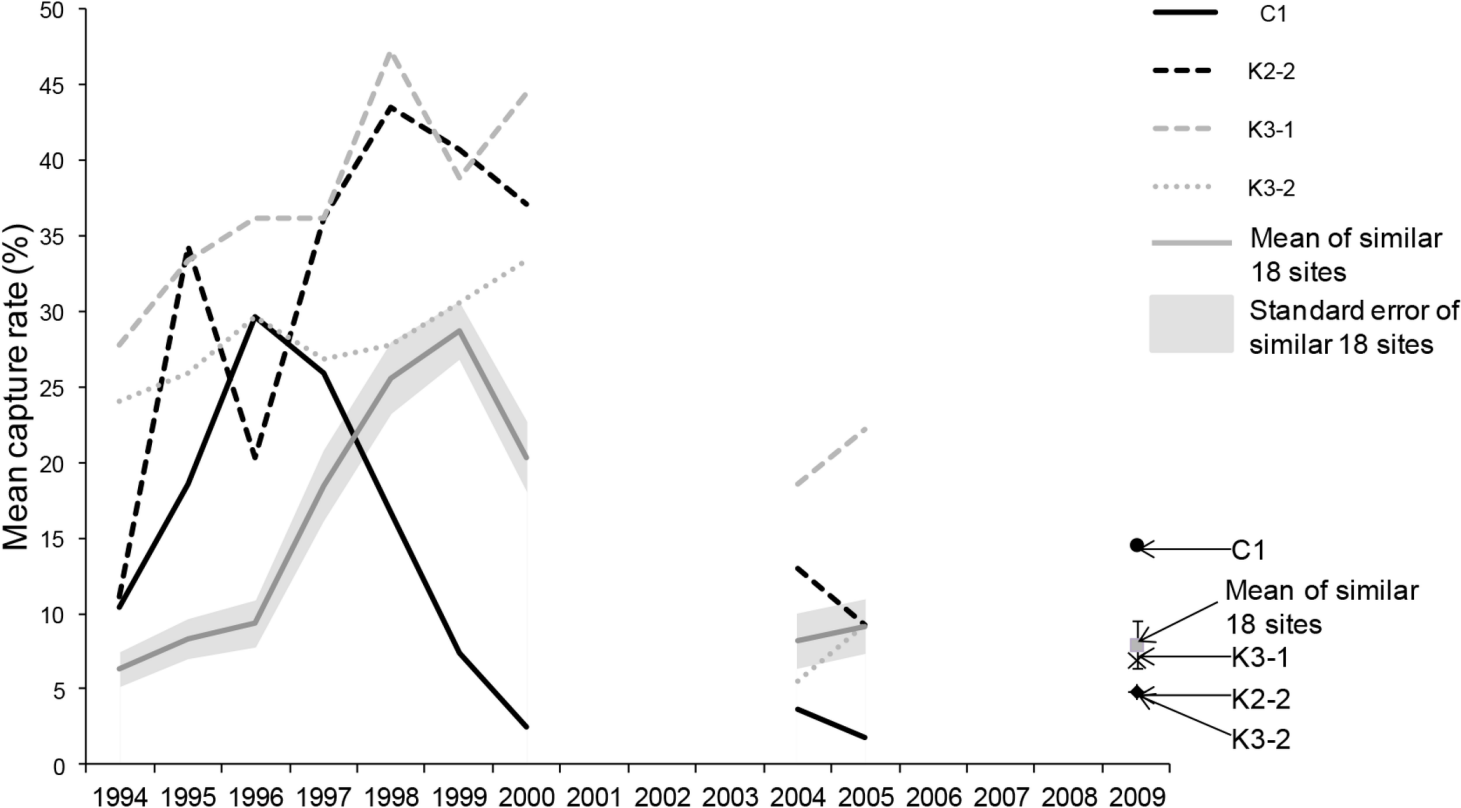
Mean capture rate at 22 grid locations between 1994 and 2009. Grey solid line represents the mean capture rate of the 18 sites that had similar abundance curve profiles and the grey shading indicates standard error. The remaining lines represent sites that were unusual either because they had different shaped abundance curves (K2-2, K3-1 and K3-2) or peaked and declined earlier than all other sites (C1). Bars associated with the mean of the similar 18 sites in 2009 indicate standard error.

The profiles of the abundance curves of woylies at 18 of the 22 grid sites followed a similar pattern over time although there was a large degree of variation in the absolute abundance between the sites each year. These 18 sites all had lower abundances between 1994 and 1996 after which there was a rapid increase with abundance peaking in either 1998 or 1999 (Fig 2). Some of the grid sites that had the greatest abundance of woylies in 1994 and before the population decline (e.g. K2-1, K2-2, K3-1 and K3-2), had some of the lowest abundances in 2009. Conversely, there were examples of sites that had lower woylie abundance in the first two years of sampling that had some of the larger abundances in 2009 (e.g. K5-4, K5-3 and K5-5). Site C1 appears to peak and begin declining earlier than any other site (peak in 1996) and had the lowest abundance of any site by 2000 (Fig 2).

**Fig 3:**
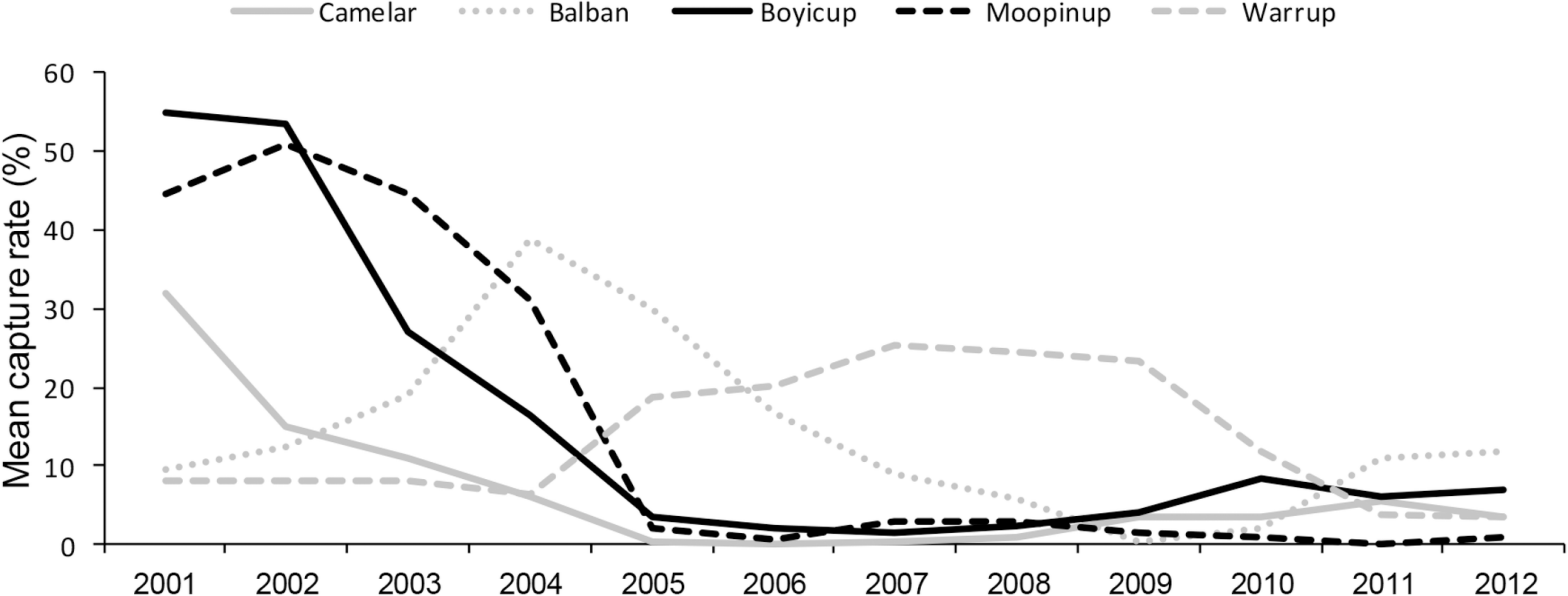
Mean capture rate at five transects in the Upper Warren between 2001 and 2012.

Three of the five transects had similar abundance curve profiles over time (Camelar, Moopinup and Boyicup). In 2001 these transects had moderate to high capture rates between 30 and 55%. Boyicup and Moopinup did not show evidence of a decline until 2003 whereas Camelar declined no later than 2002. Camelar had a slower rate of decline than the other two similar transects but by 2005, these three transects had very similar abundances of woylies (Fig 3). Balban and Warrup had different abundance patterns and both had much lower abundances of woylies than the other three transects when sampling first began in 2001. Between 2001 and 2004, Balban had an increasing abundance while the other transects were declining or remaining stable (Warrup). After its peak in 2004, Balban began to decline in abundance until 2008 when it had similar levels to that of Moopinup, Camelar and Boyicup (Fig 3). After remaining at low but stable abundances between 2001 and 2004, Warrup gradually increased and by 2007–2009 had capture rates >20%, which was an opposite trend to the other four transects. In the last year of sampling (2012) all five transects had very similar low levels of abundance (<12% capture rate). For all further analyses we grouped the three similar transects (Camelar, Moopinup and Boyicup) and analysed Balban and Warrup individually.

### Change in woylie abundance following predator control

**Fig 4:**
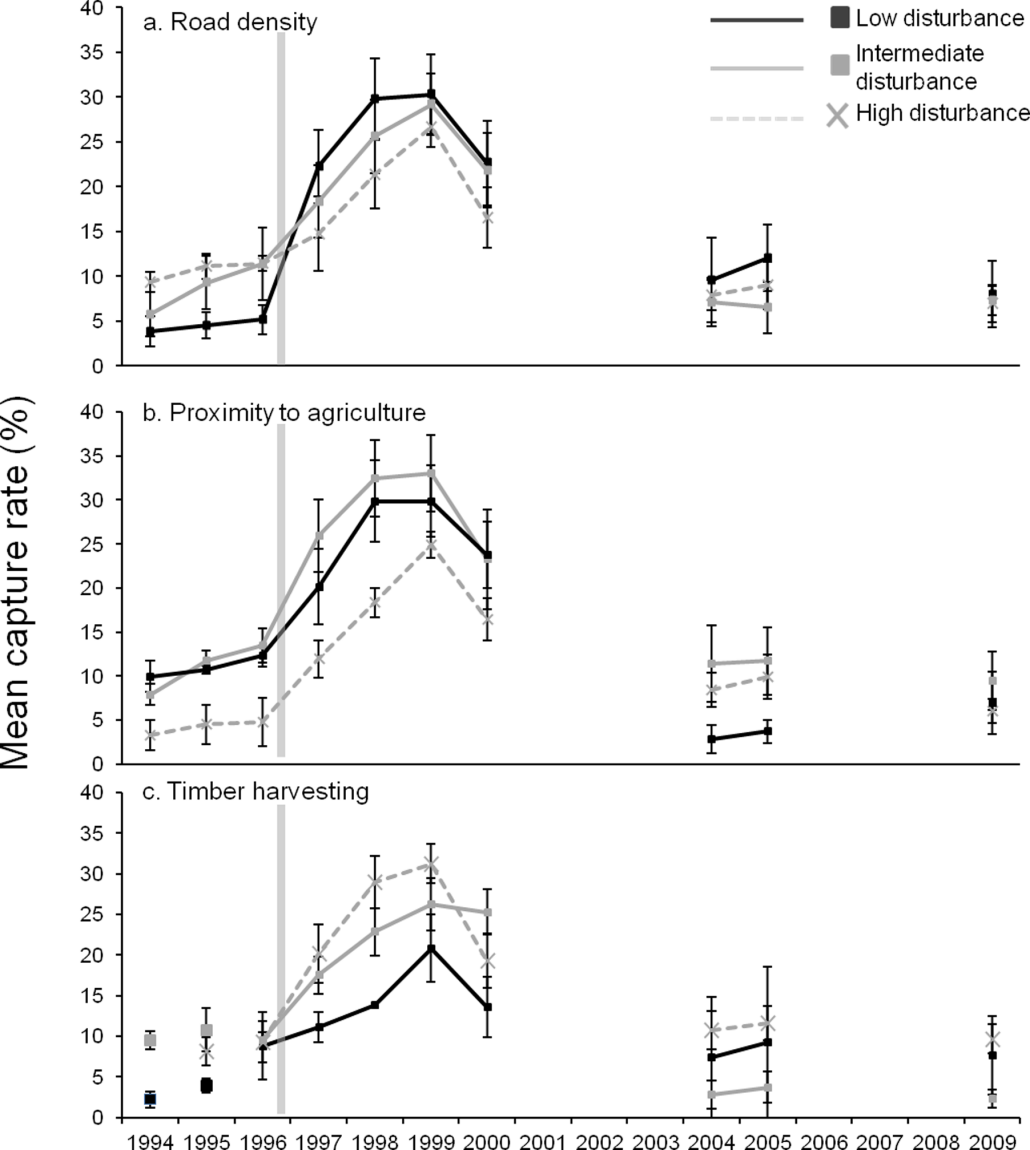
Mean capture rate of grid sites over time grouped by level of disturbance. a) Road density. Low: <1500 m, n = 6. Intermediate: 1600–2400 m, n = 6. High: >2400 m, n = 6. b) Proximity to agriculture. Low: >900 m, n = 4. Intermediate: 400–810 m, n = 6. High: <300 m, n = 8. c) Time since timber harvest. Low: 44 yrs in 1994, n = 2; 45 yrs in 1995, n = 3; 46 yrs in 1996 onwards, n = 2. Intermediate: 21–24 yrs in 1994, n = 10; 25 yrs in 1995, n = 6; 26 yrs in 1996 onward, n = 5. High: harvested in 1995 or 1996, n = 9 in 1995 and n = 11 in 1996 onwards. Transparent grey vertical line indicates time of commencement of quarterly aerial fox baiting. In Fig 3C, site classification varied between 1994 and 1996 so 1994 and 1995 are represented by data points only. In 1994, there were no sites classified as highly disturbed by timber harvesting. Bars indicate standard error.

Data collected before the implementation of invasive predator control methods was only available at grid sites and comparisons were made using only data from the 18 grid sites that had similar abundance curve patterns. Broad-scale aerial and ground fox-baiting was implemented across the Upper Warren at the end of 1996. Following this there was a rapid increase in the abundance of woylies at all but three grid sites. The magnitude of change varied between sites with different levels of disturbance. Sites with few roads had the largest change in abundance between 1996 and 1998 increasing 5.8 fold compared to 2.2 and 1.8 fold for sites with intermediate and many roads respectively (Fig 4A). Sites with varying levels of disturbance from agriculture had fairly similar increases in abundance with sites at far and intermediate distances increasing 2.4 fold and close sites 3.8 fold (Fig 4B). Sites most recently harvested had a greater increase in abundance than long unharvested sites (3.1 fold compared to 1.5 fold; Fig 4C). This may be a result of confounding with disturbance by roads, as sites with intermediate and longest times since timber harvesting also had a greater average density of roads (2026 m ± 369.0 and 2057 ± 407.6 respectively) compared to the sites that were more recently harvested (1857 ±249.1).

### Disturbance before, during and after decline

Prior to the decline (between 1994 and 1999), grid sites that were the furthest and intermediate distances from agriculture (i.e. less disturbed) had greater abundances of woylies (at least 5% higher capture rates) than sites that were close to agriculture (Fig 4B). Between 1994 and 1996, grid sites with the lowest road density had the lowest abundance of woylies but between 1996 and 1997 (following the implementation of broad-scale fox control), these sites had a rapid increase in abundance and remained the sites with the greatest abundance in all other subsequent years of sampling (though only marginally higher than sites with more roads in 2009; Fig 4A). The lower abundance of woylies at sites with few roads between 1994 and 1996 may be explained by their proximity to agriculture. On average, sites with few roads were an intermediate distance to agriculture (638 m ± 106.5) and this was similar to the average proximity to agriculture of sites with many roads (591 ± 375.4). The proximity to agriculture of sites with many roads was also widely variable and some of the sites most disturbed by roads were the least disturbed by agriculture.

Before and in the first year of the decline (1994–2000), grid sites that had the longest time since timber harvesting had the lowest abundance of woylies (Fig 4C). Again, this may be a result of confounding with road density as longer unharvested sites had a greater average density of roads. Sites that had the longest time since harvest were also on average closer to agriculture than the intermediate and recently harvested sites (362 m ±108.1 compared to 896 m ±383.4 and 657 m ±180 respectively). Between 1996 and 2009, the average capture rates of sites with the longest time since timber harvest were only based on two locations compared to ten and six locations for the recent and intermediate harvest time respectively. This meant there was less confidence in these averages as representative values for long unharvested sites.

**Fig 5:**
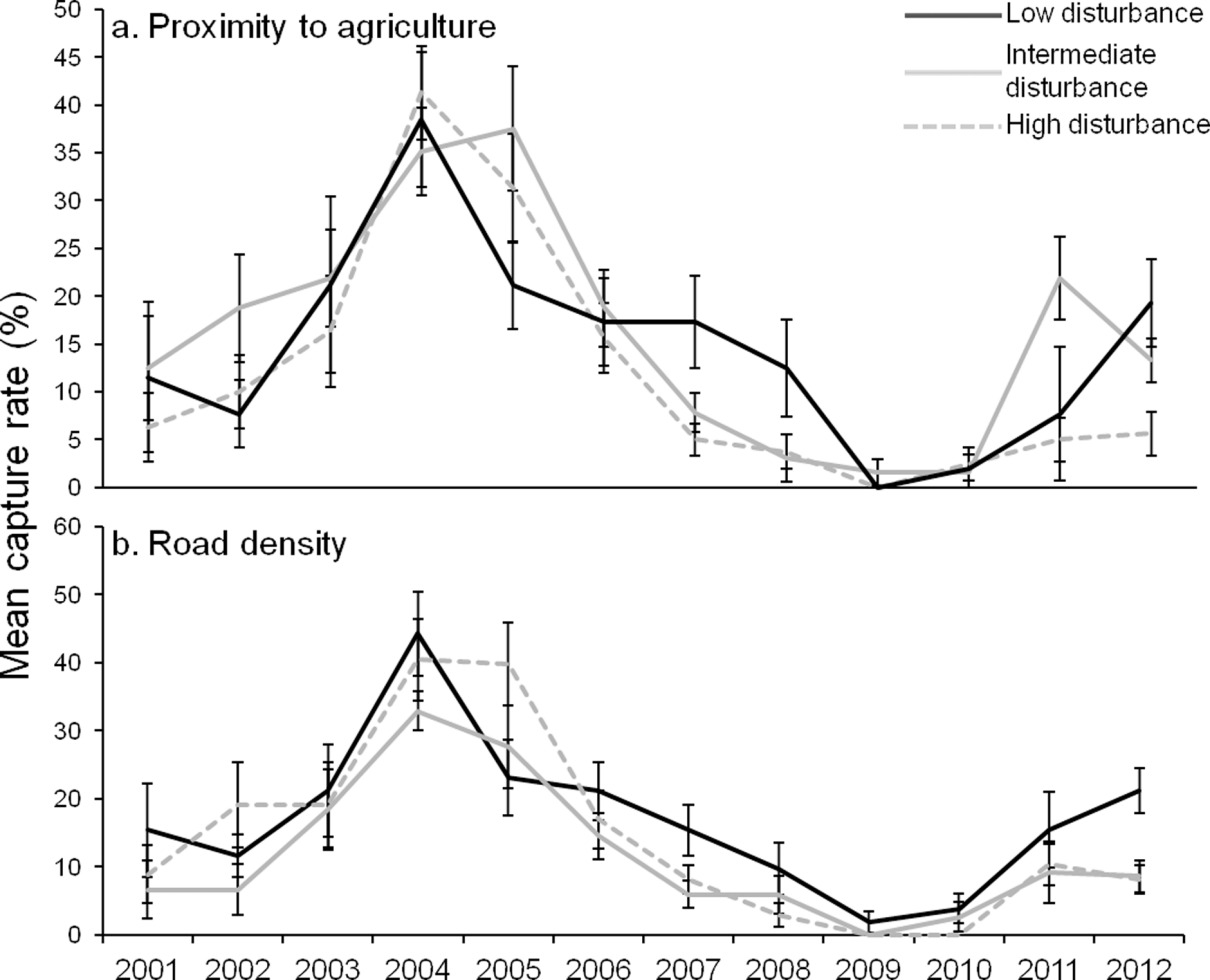
Mean capture rate of trap points at Balban transect over time grouped by level of disturbance. a) Proximity to agriculture. Low: >2000 m, n = 11. Intermediate: 1000–2000 m, n = 18. High: <1000 m, n = 22. b) Road density. Low: <1500 m, n = 16. Intermediate: 1500–2000 m, n = 16. High: >2000 m, n = 17. Bars indicate standard error.

**Fig 6:**
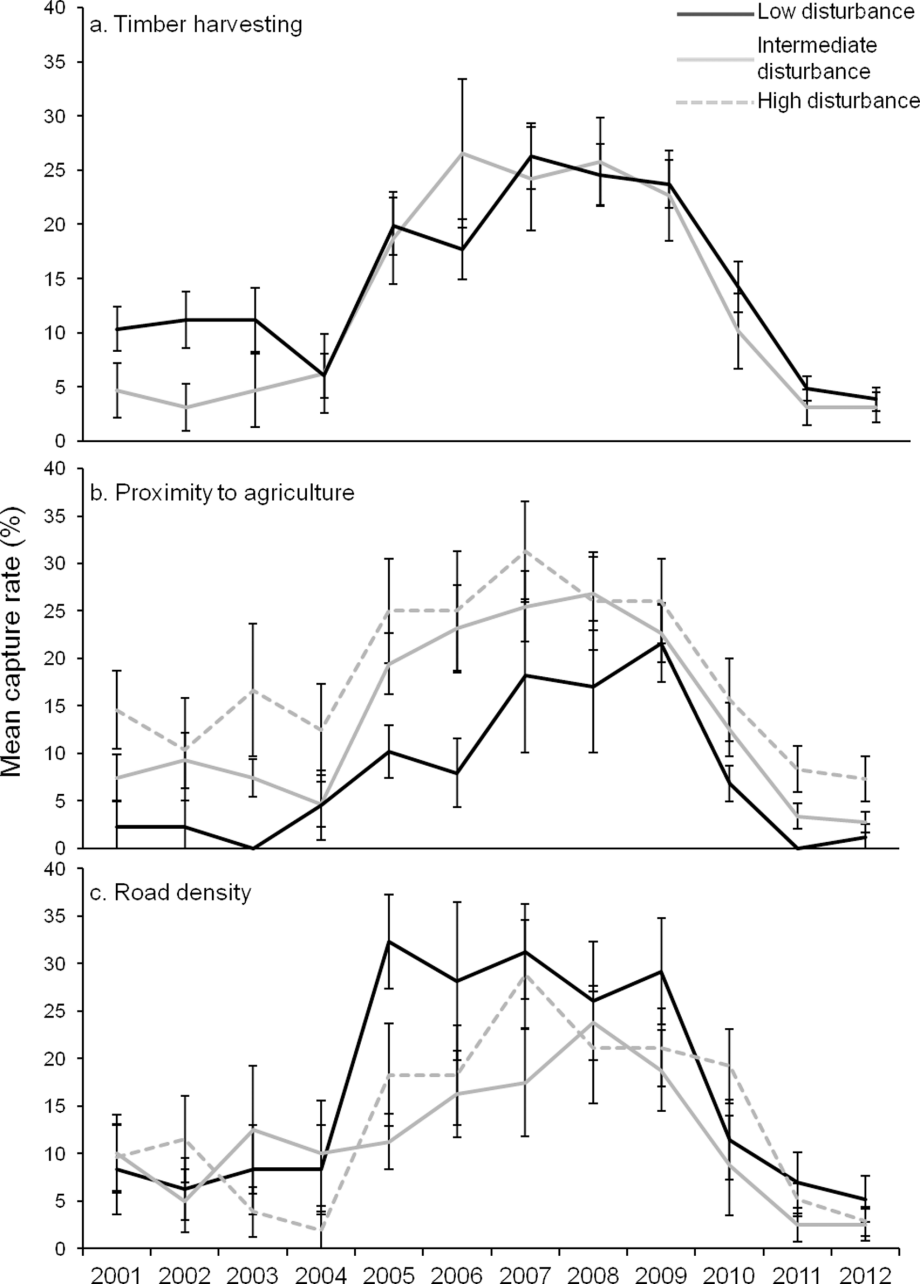
Mean capture rate of trap points at Warrup transect over time grouped by level of disturbance. a) Time since timber harvesting. Low: >42 years, n = 29. Intermediate: 22 years, n = 16. b) Proximity to agriculture. Low: >2000 m, n = 11. Intermediate: 1000–2000 m, n = 27. High: <1000 m, n = 12. c) Road density. Low: <1500 m, n = 12. Intermediate: 1500–2000 m, n = 10. High: >2500 m, n = 13. Note: there was no high disturbance category for time since timber harvesting for the Warrup transect. Bars indicate standard error.

There was no pre-decline data available for three of the transect sites (Moopinup, Camelar and Boyicup). Balban and Warrup transect appeared to be in a different stage of decline than the other transect sites so pre-decline data at these transects was categorised as data collected during peak population abundances. Balban showed no difference in the abundance of woylies before decline between sites with varying levels of agricultural or road disturbance (Fig 5; timber harvesting was not compared at Balban as there was no variation in time since last harvest). During peak abundances at Warrup, there was no difference in the abundance of woylies between sites with variable timber harvesting history (Fig 6A). Unlike the evidence from grid locations and to some extent the other transects (Moopinup, Camelar and Boyicup), throughout the sampling period at Warrup, sites far from agriculture consistently captured fewer woylies than other sites (Fig 6B). The confounding factor of road density may account for the unexpectedly low capture rates at sites far from agriculture as sites at intermediate and far distances from agriculture also had higher average road densities compared to sites close to agriculture (2122 m (± 157.5) and 2079 m (± 60.2) respectively compared to 1886 m (± 248.7)). Between 2005 and 2009 when the abundance of woylies at Warrup was at its highest, sites with fewer roads captured more woylies than sites with a greater density of roads (although only significantly more in 2005; Fig 6C).

**Fig 7:**
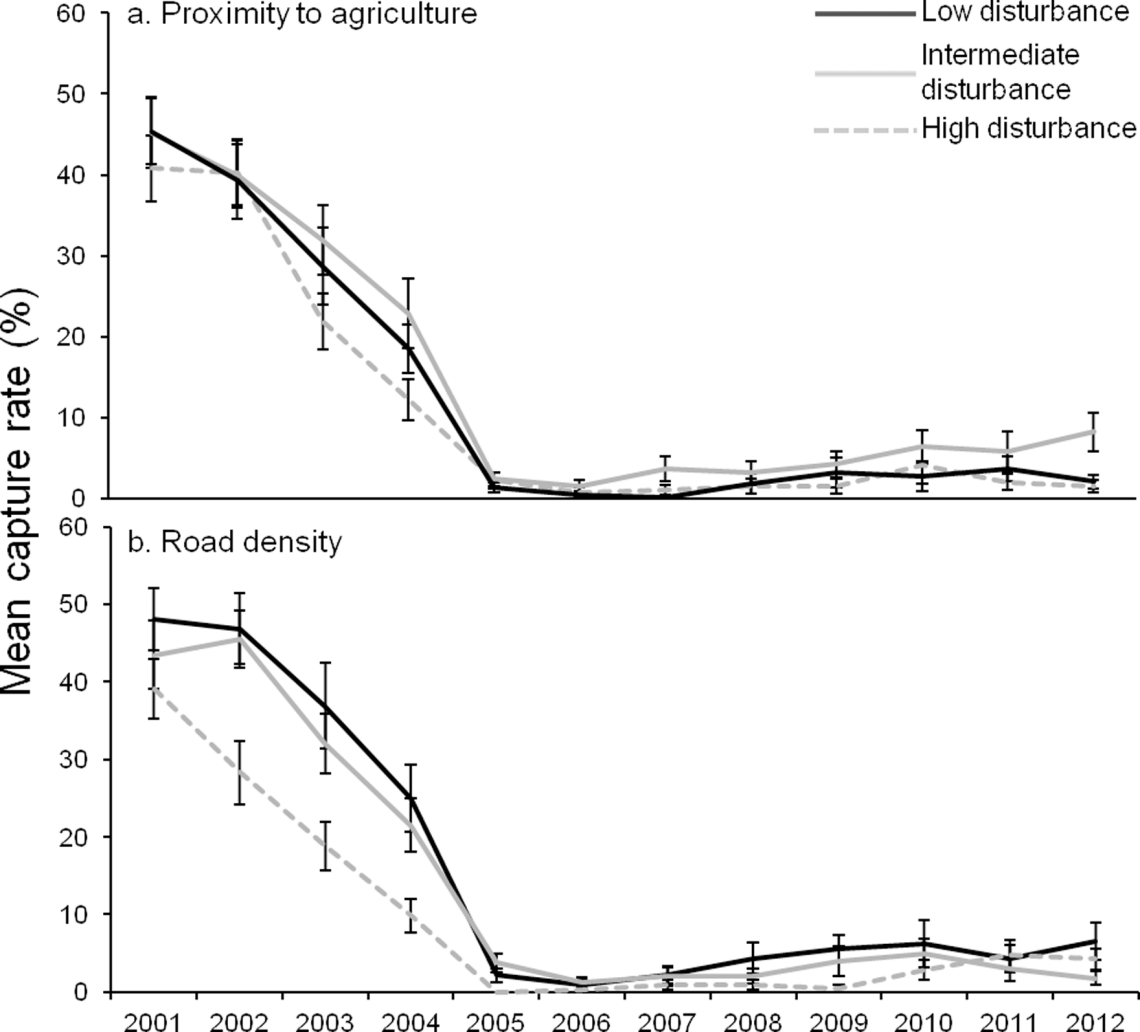
Mean capture rate of the Boyicup, Camelar and Moopinup transect trap points over time grouped by level of disturbance. a) Proximity to agriculture. Low: >2000 m, n = 47. Intermediate: 1000–2000 m, n = 47. High: <1000 m, n = 49. b) Road density. Low: <1200 m, n = 40. Intermediate: 1200–1800 m, n = 50. High: >1800 m, n = 53. Bars indicate standard error.

During the decline, there was little difference in the abundance of woylies at Camelar, Moopinup and Boyicup transects when comparing agricultural disturbance but there was some evidence that sites closer to agriculture had a steeper rate of decline between 2002 and 2005 (Fig 7A). Within these three transects, sites that had the greatest road density had lower woylie abundance during the population decline (between 5 and 10% lower capture rates; Fig 7B). During a declining trend at Balban transect (2005–2008), there was similar evidence that sites further from agriculture and those with fewer roads had a greater abundance of woylies (Fig 5). During this time (2007–2008), sites at Balban that were far from agriculture declined at a slower rate than those closer to agriculture and caught 12 and 9% more woylies than sites close to agriculture during those years respectively (Fig 5A). In 2007, sites with fewer roads also had a greater abundance of woylies (Fig 5B). There was not enough available data collected during the decline phase at Warrup (only one sample year was categorised as a declining state), no data available during the decline at grid sites and no variation in the timber harvesting history at the remaining four transect locations to compare disturbance between these sites during the decline phase.

After the decline, there was little difference in the abundance of woylies between sites with varying levels of disturbance at most grid and transect locations (Fig 4 and Fig 7). Balban transect showed the same pattern but during a possible start of a recovery in 2011–2012, sites with intermediate and low level agricultural disturbance and low level road disturbance had significantly higher abundances of woylies than sites that were highly disturbed (Fig 5). In 2012, sites at Balban with few roads caught over twice the number of woylies compared to sites with many roads (21 and 8% capture rates respectively; Fig 5B). Warrup transect had two periods of decline (2001–2003 and 2011–2012) and during the first decline phase, there was a greater abundance of woylies in long unharvested sites (Fig 6A). There was a greater abundance of woylies at Warrup sites close to agriculture (potentially a result of confounding with road density) and little difference between sites of varying levels of road disturbance during declined periods (Fig 6B and 6C respectively).

**Fig 8:**
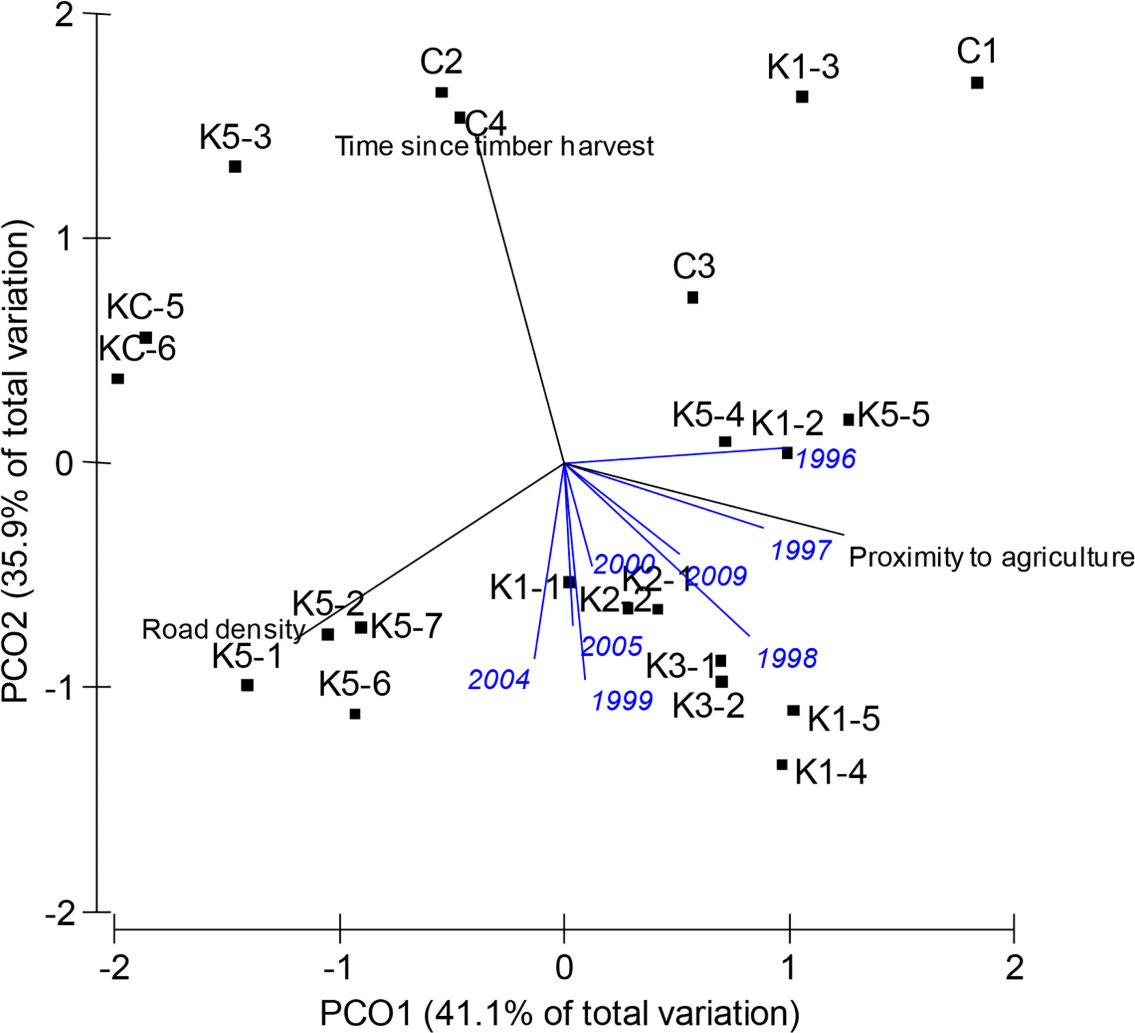
Analysis of Principal Coordinates (PCO) based on the time since timber harvesting, proximity to agriculture and road density of each of 22 grids between 1996 and 2009. Blue vector overlays represent Pearson’s correlation coefficients of mean capture rate during a particular year against the PCO axes. Black vector overlays represent Pearson’s correlation coefficients of these variables against the PCO axes. Vector length indicates strength of correlation. The analysis was based on Euclidian distances calculated from square-root transformed values.

**Fig 9:**
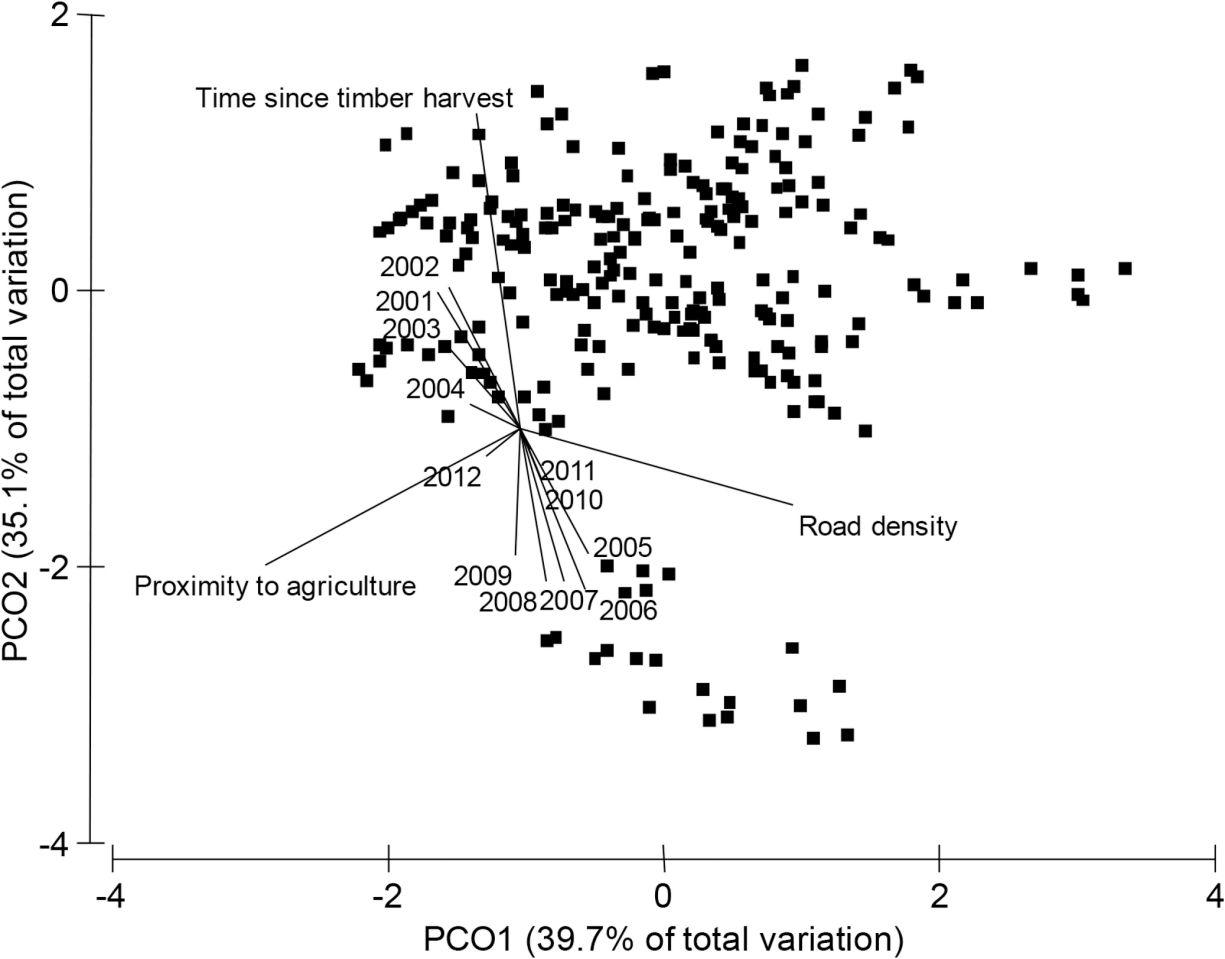
Analysis of Principal Coordinates (PCO) based on the time since timber harvesting, proximity to agriculture and road density of 242 trap points along five transects in the Upper Warren (Boyicup, Moopinup, Camelar, Warrup and Balban). Vector overlays labelled with year represent Pearson’s correlation coefficients of mean capture rate during a particular year against the PCO axes. Vector overlays labelled with landscape variables represent Pearson’s correlation coefficients of these variables against the PCO axes. Vector length indicates strength of correlation. The analysis was based on Euclidian distances calculated from square-root transformed values.

To further understand how woylie abundance related to the three disturbance factors over time, we conducted a PCO based on the time since timber harvest, proximity to agriculture and road density of the 22 grids (Fig 8) and five transects (Fig 9). We then overlaid vectors indicating the association between woylie abundance during a particular year and the PCO axes. For the grid sites, separate PCO analyses were conducted on 1994 and 1995 data because of harvesting events during these years (see S2 Fig for 1994 and 1995 PCO plots). Between 1994 and 1997, woylie abundance at grid sites was positively associated with distance to agriculture and often negatively associated with road density (Fig 8 and S2 Fig). In 1998 there was a slight change where sites that had the greatest abundance of woylies were those that were far from agriculture but also had the shortest time since timber harvesting. Between 1999 and 2005, abundance was greatest in sites with the shortest time since timber harvesting with variable distances to agriculture. In the final year of sampling (2009), a similar pattern was present to earlier years (1997 and 1998), where sites with the greatest woylie abundance were further from agriculture and had shorter times since timber harvesting.

PCO of the five transect sites showed that in the years 2001 and 2002, woylie abundance was greatest at sites with fewer roads (Fig 9). Contrary to the grid locations, during this time, woylie abundance was positively associated with time since timber harvesting. In 2003 and 2004 there was a slight change where the greatest woylie abundance was at sites that were long unharvested and with fewer roads, but at some sites also further from agriculture. There was a rapid change in 2005 where sites that had the shortest time since timber harvesting and the lowest abundances in 2001/02, had the greatest abundance in the years 2005–2009. These sites had some of the largest distances to agriculture and were all from the Warrup transect. There was not as strong a trend in the abundance of woylies between 2010 and 2012, most likely because all sites caught very few woylies. The rapid change in the characteristics of sites that had the greatest abundance of woylies in the years 2005–2009 reflects the different stages of decline occurring between Warrup transect (potentially beginning a recovery in 2005 and increasing abundance) and the other four transects (decreasing abundance at that time; Fig 3).

## Discussion

This study provides evidence that the distribution and abundance of woylies in the Upper Warren region over time is related to the level of landscape disturbance, specifically the degree of fragmentation by roads and proximity to agriculture. Landscapes disturbed by agriculture and roads might be less robust during times of environmental change, more susceptible to threats and generally support lower abundances of woylies. This is similar to findings in other studies of invertebrates, amphibians and small mammals in forested areas where species abundance was lower in more disturbed sites [39–42]. Prior to the implementation of broad scale fox control and during the subsequent increase in woylie abundance in the following three years, sites that were the furthest from agriculture supported a greater abundance of woylies. During the population decline, there was evidence that these sites declined at a slower rate. Sites with fewer roads also had a greater abundance of woylies but this was not the case prior to the implementation of broad scale fox control. This was unexpected but is likely to be a result of confounding with proximity to agriculture. The impact of disturbance by roads and agriculture on woylies was most important during times of population change and less significant during times of low and peak abundances. This is supported by the exploratory regression analyses, where significant relationships between landscape disturbance and woylie abundance were present during but not before or after population decline. Post population decline when woylie abundance across the Upper Warren was at its lowest, landscape disturbance by roads and agriculture made little difference to the abundance of woylies between sites. It is possible, particularly in terms of the transect locations, that low overall capture rates following the population decline may have contributed to a lack of observable differences in the abundance of woylies between sites.

It is less clear how woylie abundance relates to timber harvesting. At times when the population in the Upper Warren was at its largest, the most abundant grid sites were also the most recently harvested. Other than during three consecutive years, data from Warrup transect (the only transect that had variable harvesting history and therefore the only transect where timber harvesting was compared) showed no clear difference in the abundance of woylies based on timber harvesting history. Data from long unharvested grid sites was only available from two locations for a large portion of the years surveyed (1996 to 2009) and these sites had higher road densities and were closer to agriculture. Fewer available long unharvested grid sites reduced the confidence in the value of these sites as representative of long unharvested areas. Findings by Wayne *et al*. [21] and Wayne *et al*. [22] suggest no negative response of woylie number to timber harvesting, the latter also finding that population declines in recently harvested areas were less than long unharvested sites. Wayne *et al*. [21] states that sites classified as ‘never harvested’ confounded with fox-baiting history and six out of the eight sites had never been baited. It is possible that factors such as fox-baiting history, road density and proximity to agriculture were confounding factors in these studies. It may be that woylies are unaffected by selective timber harvesting as they are a ground-dwelling, generalist species and disturbance by harvesting does not necessarily also mean a loss of habitat. As it stands, additional research is required to clarify the relationship between woylie abundance and timber harvesting.

As a result of utilising a dataset collected as part of a fauna monitoring program and not designed to test the impact of landscape disturbance on woylie abundance, this paper is largely descriptive and has generated hypotheses that warrant further testing. A multivariate approach to the analysis of the associations between landscape disturbance and woylie abundance would be ideal but this was difficult to achieve because of missing data from years that were not sampled. Key features of future experiments to further test the impact of disturbance or the factors associated with woylie abundance more generally, include a fully replicated design, repeated measures without missing data, an estimate of the abundance of invasive predators and consideration of finer scale habitat characteristics of experimental sites.

Agriculture can disturb natural landscapes by elevating the abundance of invasive predators [43, 44] and roads can facilitate the movement of these species [11–13]. Cats and foxes have been recorded ranging widely from agriculture into forested areas and foxes found several kilometres from agricultural land have been found with agricultural stock in their guts [8]. In a recent trial study investigating the use of camera traps for monitoring foxes and cats, across 132 sites both species were seen on cameras next to tracks significantly more often than off tracks (31 foxes on tracks compared to twelve off tracks and twelve cats on tracks compared to zero off tracks; Wayne *et al*. [8]). Both foxes and cats are strongly associated with woylie mortality and abundance in south-western Australia [21, 45–47] and so understanding the factors associated with their abundance will be an important part of woylie conservation in the future.

Agricultural land and roads can also be indicative of disturbance unrelated to invasive predator density [2, 48, 49]. Less disturbed sites may be better able to cope with threats and environmental change [1, 50, 51]. In the current study, sites with few roads had a greater rate of increase in abundance after the implementation of broad scale fox control than sites with many and this suggests that these sites provide higher quality habitat for woylies. This could be a result of habitat degradation in sites highly dissected by roads, population fragmentation [29], weed species invasion, increased mortality of woylies from road collisions or a reduced amount of available habitat [2].

There was some evidence that sites with the greatest abundance pre-population decline had some of the lowest abundances post decline. This pattern might indicate that higher density sites were more vulnerable to decline and may support one of the current theories identifying disease as a possible causative agent of the decline [30, 33, 34, 52, 53]. Disease transfer rate may be greater in high density populations [54, 55] resulting in a higher prevalence of the disease overall compared to low density populations [56, 57]. If this were the case in the Upper Warren, it may be that lower density sites before the decline had lower disease prevalence and so had a larger number of individuals remaining after the decline that were uninfected (or were more recently infected and had not developed chronic disease). These sites may have been able to recover sooner because they had more individuals remaining, were able to reproduce faster and increase in abundance.

## Conclusion

In terms of the future conservation of woylies, the results of this study suggest that sites of varying levels of disturbance can support woylie populations, but sites disturbed by agriculture and roads might be less robust during environmental change, more susceptible to threats and generally support lower abundances of woylies. Strategies that reduce the impact of disturbance on woylie populations could include the rationalisation of forest tracks and roads in conservation areas with less valuable roads closed and rehabilitated. The acquisition of private property in areas of high conservation value to consolidate contiguous habitat is another strategy that could be beneficial. Reducing the impact of disturbance in the Upper Warren region could improve the resilience of this critically important woylie population during future environmental change.

## Supporting Information

S1 Fig **Scatter plot of woylie capture rate during population decline and the a) proximity to agriculture, b) road density and c) time since timber harvesting at study sites across the Upper Warren.** Trend lines indicating the direction of the relationship between woylie capture rate and each disturbance factor are included.
(DOCX)

S2 Fig **Analysis of Principal Coordinates (PCO) based on the time since timber harvesting, proximity to agriculture and road density of each of 22 grids in a) 1994 and b) 1995.** Vector overlays labelled with year represent Pearson’s correlation coefficients of mean capture rate during that year against the PCO axes. Vector overlays labelled with landscape variables represent Pearson’s correlation coefficients of these variables against the PCO axes. Vector length indicates strength of correlation. The analysis was based on Euclidian distances calculated from square-root transformed values.
(DOCX)

S1 Table Total trap effort (nights) for each grid and transect site according to year. (DOCX)

S2 Table Results of correlations between disturbance factors and regression analyses between disturbance factors and woylie capture rate at sites in the Upper Warren before, during and after population decline. Values in the Road density and Proximity to agriculture columns are correlations with the other disturbance factors. Values presented in the Before, During and After decline columns are R^2^ values with associated P-values. The regression analysis was conducted by comparing the abundance of woylies at sites before, during and after the population decline with the level of disturbance at those sites. * indicates significant P-value.
(DOCX)
