# Supplementary material for: Temporal Patterns in the Abundance of a Critically Endangered Marsupial Relates to Disturbance by Roads and Agriculture
Source: PLoS One. 2016 Aug 8;11(8):e0160790. doi: 10.1371/journal.pone.0160790 (PMC4976897; doi:10.1371/journal.pone.0160790)
Supplement: S1 Fig — Scatter plot of woylie capture rate during population decline and the a) proximity to agriculture, b) road density and c) time since timber harvesting at study sites across the Upper Warren. Trend lines indicating the direction of the relationship between woylie capture rate and each disturbance factor are included. (DOCX) [file pone.0160790.s001.docx]

**Figure S1**. Scatter plot of woylie capture rate during population decline and the a) proximity to agriculture, b) road density and c) time since timber harvesting at study sites across the Upper Warren. Trend lines indicating the direction of the relationship between woylie capture rate and each disturbance factor are included.
