# Supplementary material for: Temporal Patterns in the Abundance of a Critically Endangered Marsupial Relates to Disturbance by Roads and Agriculture
Source: PLoS One. 2016 Aug 8;11(8):e0160790. doi: 10.1371/journal.pone.0160790 (PMC4976897; doi:10.1371/journal.pone.0160790)
Supplement: S2 Fig — Analysis of Principal Coordinates (PCO) based on the time since timber harvesting, proximity to agriculture and road density of each of 22 grids in a) 1994 and b) 1995. Vector overlays labelled with year represent Pearson’s correlation coefficients of mean capture rate during that year against the PCO axes. Vector overlays labelled with landscape variables represent Pearson’s correlation coefficients of these variables against the PCO axes. Vector length indicates strength of correlation. The analysis was based on Euclidian distances calculated from square-root transformed values. (DOCX) [file pone.0160790.s002.docx]

**Figure S2**. Analysis of Principal Coordinates (PCO) based on the time since timber harvesting, proximity to agriculture and road density of each of 22 grids in a) 1994 and b) 1995. Vector overlays labelled with year represent Pearson’s correlation coefficients of mean capture rate during that year against the PCO axes. Vector overlays labelled with landscape variables represent Pearson’s correlation coefficients of these variables against the PCO axes. Vector length indicates strength of correlation. The analysis was based on Euclidian distances calculated from square-root transformed values.
