# Supplementary material for: Temporal Patterns in the Abundance of a Critically Endangered Marsupial Relates to Disturbance by Roads and Agriculture
Source: PLoS One. 2016 Aug 8;11(8):e0160790. doi: 10.1371/journal.pone.0160790 (PMC4976897; doi:10.1371/journal.pone.0160790)
Supplement: S1 Table — (DOCX) [file pone.0160790.s003.docx]

**Table S1**. Total trap effort (nights) for each grid and transect site according to year.

| Site | Design | 1994 | 1995 | 1996 | 1997 | 1998 | 1999 | 2000 | 2001 | 2002 | 2003 | 2004 | 2005 | 2006 | 2007 | 2008 | 2009 | 2010 | 2011 | 2012 |
| --- | --- | --- | --- | --- | --- | --- | --- | --- | --- | --- | --- | --- | --- | --- | --- | --- | --- | --- | --- | --- |
| C1 | Grid | 153 | 135 | 108 | 108 | 108 | 108 | 81 | 0 | 0 | 0 | 54 | 54 | 0 | 0 | 0 | 144 | 0 | 0 | 0 |
| C2 | Grid | 153 | 135 | 108 | 108 | 108 | 108 | 81 | 0 | 0 | 0 | 54 | 54 | 0 | 0 | 0 | 144 | 0 | 0 | 0 |
| C3 | Grid | 108 | 135 | 108 | 108 | 108 | 108 | 81 | 0 | 0 | 0 | 54 | 54 | 0 | 0 | 0 | 144 | 0 | 0 | 0 |
| C4 | Grid | 108 | 135 | 108 | 108 | 108 | 117 | 81 | 0 | 0 | 0 | 54 | 54 | 0 | 0 | 0 | 144 | 0 | 0 | 0 |
| K1-1 | Grid | 108 | 108 | 108 | 108 | 108 | 108 | 81 | 0 | 0 | 0 | 54 | 54 | 0 | 0 | 0 | 144 | 0 | 0 | 0 |
| K1-2 | Grid | 108 | 108 | 108 | 108 | 108 | 108 | 81 | 0 | 0 | 0 | 54 | 54 | 0 | 0 | 0 | 144 | 0 | 0 | 0 |
| K1-3 | Grid | 108 | 108 | 108 | 108 | 108 | 108 | 81 | 0 | 0 | 0 | 54 | 54 | 0 | 0 | 0 | 144 | 0 | 0 | 0 |
| K1-4 | Grid | 108 | 135 | 108 | 108 | 108 | 108 | 81 | 0 | 0 | 0 | 54 | 54 | 0 | 0 | 0 | 144 | 0 | 0 | 0 |
| K1-5 | Grid | 108 | 135 | 108 | 108 | 108 | 108 | 81 | 0 | 0 | 0 | 54 | 54 | 0 | 0 | 0 | 144 | 0 | 0 | 0 |
| K2-1 | Grid | 54 | 153 | 108 | 108 | 108 | 108 | 81 | 0 | 0 | 0 | 54 | 54 | 0 | 0 | 0 | 144 | 0 | 0 | 0 |
| K2-2 | Grid | 108 | 108 | 108 | 108 | 108 | 108 | 81 | 0 | 0 | 0 | 54 | 54 | 0 | 0 | 0 | 144 | 0 | 0 | 0 |
| K3-1 | Grid | 108 | 108 | 108 | 108 | 108 | 108 | 81 | 0 | 0 | 0 | 54 | 54 | 0 | 0 | 0 | 144 | 0 | 0 | 0 |
| K3-2 | Grid | 108 | 108 | 108 | 108 | 108 | 108 | 81 | 0 | 0 | 0 | 54 | 54 | 0 | 0 | 0 | 144 | 0 | 0 | 0 |
| K5-1 | Grid | 153 | 126 | 108 | 108 | 108 | 108 | 81 | 0 | 0 | 0 | 54 | 54 | 0 | 0 | 0 | 144 | 0 | 0 | 0 |
| K5-2 | Grid | 153 | 126 | 108 | 108 | 108 | 108 | 81 | 0 | 0 | 0 | 54 | 54 | 0 | 0 | 0 | 144 | 0 | 0 | 0 |
| K5-3 | Grid | 153 | 135 | 108 | 108 | 108 | 108 | 81 | 0 | 0 | 0 | 54 | 54 | 0 | 0 | 0 | 144 | 0 | 0 | 0 |
| K5-4 | Grid | 126 | 135 | 108 | 108 | 108 | 108 | 81 | 0 | 0 | 0 | 54 | 54 | 0 | 0 | 0 | 144 | 0 | 0 | 0 |
| K5-5 | Grid | 126 | 135 | 108 | 108 | 108 | 108 | 81 | 0 | 0 | 0 | 54 | 54 | 0 | 0 | 0 | 144 | 0 | 0 | 0 |
| K5-6 | Grid | 126 | 135 | 144 | 144 | 108 | 108 | 81 | 0 | 0 | 0 | 54 | 54 | 0 | 0 | 0 | 144 | 0 | 0 | 0 |
| K5-7 | Grid | 126 | 135 | 144 | 144 | 108 | 108 | 81 | 0 | 0 | 0 | 54 | 54 | 0 | 0 | 0 | 144 | 0 | 0 | 0 |
| KC-5 | Grid | 216 | 270 | 216 | 216 | 216 | 216 | 135 | 0 | 0 | 0 | 108 | 54 | 0 | 0 | 0 | 288 | 0 | 0 | 0 |
| KC-6 | Grid | 216 | 270 | 216 | 216 | 216 | 216 | 135 | 0 | 0 | 0 | 108 | 54 | 0 | 0 | 0 | 288 | 0 | 0 | 0 |
| Balban | Transect | 0 | 0 | 0 | 0 | 0 | 0 | 0 | 200 | 200 | 200 | 400 | 200 | 400 | 400 | 400 | 400 | 200 | 200 | 400 |
| Boyicup | Transect | 0 | 0 | 0 | 0 | 0 | 0 | 0 | 200 | 200 | 200 | 200 | 400 | 400 | 200 | 200 | 200 | 200 | 200 | 200 |
| Camelar | Transect | 0 | 0 | 0 | 0 | 0 | 0 | 0 | 200 | 200 | 200 | 200 | 200 | 400 | 400 | 200 | 200 | 200 | 200 | 400 |
| Moopinup | Transect | 0 | 0 | 0 | 0 | 0 | 0 | 0 | 200 | 200 | 200 | 200 | 400 | 400 | 200 | 200 | 200 | 200 | 200 | 200 |
| Warrup | Transect | 0 | 0 | 0 | 0 | 0 | 0 | 0 | 200 | 200 | 200 | 200 | 400 | 400 | 400 | 400 | 400 | 400 | 600 | 400 |
