# Supplementary material for: Temporal Patterns in the Abundance of a Critically Endangered Marsupial Relates to Disturbance by Roads and Agriculture
Source: PLoS One. 2016 Aug 8;11(8):e0160790. doi: 10.1371/journal.pone.0160790 (PMC4976897; doi:10.1371/journal.pone.0160790)
Supplement: S2 Table — Values in the Road density and Proximity to agriculture columns are correlations with the other disturbance factors. Values presented in the Before, During and After decline columns are R2 values with associated P-values. The regression analysis was conducted by comparing the abundance of woylies at sites before, during and after the population decline with the level of disturbance at those sites. * indicates significant P-value. (DOCX) [file pone.0160790.s004.docx]

**Table S2**. Results of correlations between disturbance factors and regression analyses between disturbance factors and woylie capture rate at sites in the Upper Warren before, during and after population decline. Values in the Road density and Proximity to agriculture columns are correlations with the other disturbance factors. Values presented in the Before, During and After decline columns are R^2^ values with associated P-values. The regression analysis was conducted by comparing the abundance of woylies at sites before, during and after the population decline with the level of disturbance at those sites. * indicates significant P-value.

| **Disturbance factor** | **Road density** | **Proximity to agriculture** | **Before** | **During** | **After** |
| --- | --- | --- | --- | --- | --- |
| **Road density** | - | -0.24 | 0.01 (P = 0.24) | 0.06 (P = 0.0006)* | 0.003 (P = 0.44) |
| **Proximity to agriculture** | - | - | 0.02 (P = 0.07) | 0.04 (P = 0.002)* | 0.01 (P = 0.26) |
| **Time since timber harvesting** | -0.25 | 0.04 | 0.001 (P = 0.61) | 0.07 (P = 0.0001)* | 0.01 (P = 0.16) |
